# The Role of Dihydroresveratrol in Enhancing the Synergistic Effect of *Ligilactobacillus salivarius* Li01 and Resveratrol in Ameliorating Colitis in Mice

**DOI:** 10.34133/2022/9863845

**Published:** 2022-06-14

**Authors:** Yiqiu Fei, Shuobo Zhang, Shengyi Han, Bo Qiu, Yanmeng Lu, Weixing Huang, Fang Li, Deying Chen, Björn Berglund, Hang Xiao, Lanjuan Li, Mingfei Yao

**Affiliations:** ^1^State Key Laboratory for Diagnosis and Treatment of Infectious Diseases, National Clinical Research Center for Infectious Diseases, Collaborative Innovation Center for Diagnosis and Treatment of Infectious Diseases, The First Affiliated Hospital, Zhejiang University School of Medicine, Hangzhou 310003, China; ^2^Jinan Microecological Biomedicine Shandong Laboratory, Jinan 250021, China; ^3^Zhejiang Tongchuang Yuecheng Health Science and Technology Co., Ltd., Shaoxing, China; ^4^Cardiometabolic Genomics Program, Division of Cardiology, Department of Medicine, Columbia University Irving Medical Center, 630 W 168th St, P&S10-401, New York, NY 10032, USA; ^5^Department of Food Science, University of Massachusetts, Amherst, Massachusetts 01003, USA; ^6^Department of Biomedical and Clinical Sciences, Linköping University, Linköping, Sweden; ^7^Zhejiang University, Department of Food Science and Nutrition, Zhejiang Key Laboratory for Agro-Food Processing, Fuli Institute of Food Science, Zhejiang R & D Center for Food Technology and Equipment, Hangzhou 310058, China

## Abstract

Currently approved therapeutical strategies for inflammatory bowel diseases (IBD) suffer from variable efficacy and association with risk of serious side effects. Therefore, efforts have been made in searching for alternative therapeutics strategies utilizing gut microbiota manipulation. In this study, we show that the probiotic strain *Ligilactobacillus salivarius* Li01 (Li01) and the phytochemical prebiotic resveratrol (RSV) have synergistic effect in ameliorating colitis in mice. Oral coadministration of Li01 (10^9^ CFU/d) and RSV (1.5 g/kg/d) promoted restoration of various inflammatory injuries and gut microbiota composition, exhibiting a favorable anti-inflammatory effect in DSS-induced colitis mice. The combination treatment was associated with reductions in the levels of proinflammatory cytokines IL-1*β* and IL-6 and increases in the levels of the anti-inflammatory cytokine IL-17A in mouse serum. Moreover, the combination treatment was found to alter the composition and metabolism of the gut microbiota, especially influencing the production of short chain fatty acids and anti-inflammatory related molecules. The mechanism underlying the improved anti-inflammatory effect from the RSV and Li01 combination treatment was found to be associated with the environmental sensor mammalian aryl hydrocarbon receptor (AHR) and tryptophan metabolism pathway. Administration of RSV in combination with Li01 in different mouse model led to enhanced conversion of RSV into metabolites, including dihydroresveratrol (DHR), resveratrol-sulfate, and resveratrol-glucuronide. DHR was found to be the dominant metabolite of RSV in conventional and colitis mice. An increased DHR/RSV ratio was confirmed to activate AHR and contribute to an enhanced anti-inflammatory effect. DHR is considered as a potential AHR ligand. The DHR/RSV ratio also affected the serotonin pathway by controlling the expression of Tph1, SERT, and 5-HT_7_R leading to amelioration of colitis in mice. Our data suggest that treatment with a combination of Li01 and RSV has potential as a therapeutic strategy for IBD; further investigation of this combination in clinical settings is warranted.

## 1. Introduction

Inflammatory bowel disease (IBD), a group of chronic inflammatory diseases, includes ulcerative colitis (UC) and Crohn's disease (CD), driven by a combination of environmental, genetic, microbial, and immune factors [1]. The main symptoms of IBD include abdominal pain, diarrhea, and weight loss, which seriously affected the quality of life of the patient. At present, although the pathogenesis of IBD is still unknown, many studies have suggested that intestinal microbial dysbiosis and microbial metabolic disorders are closely associated with IBD development [2]. For instance, a high-salt diet resulted in a decrease in the abundance of *Lactobacillus* spp. and butyrate metabolism in the gut, which exacerbates intestinal inflammation in return [3]. Therefore, many researches highlight the approaches (such as probiotics, prebiotics, and postbiotics) and their prospects in modulating the gut microbiota in ameliorating the diseases.

Probiotics have a symbiotic relationship with the host. They can obstruct pathogenic bacteria from colonizing the gut and protect the intestinal barrier function [4]. In addition, their digestive products, short chain fatty acids (SCFAs), are the main energy source for intestinal cells and can regulate fat metabolism and prevent intestinal epithelial damage [5]. Probiotics may also be involved in the metabolism of some compounds, which may enhance their function in the body. For instance, *Lactobacillus* spp. participate in the metabolism of plant glycosides and externalizes their bioactive phytochemicals by encoding enzyme systems, making glycosides easy to be absorbed and thus facilitating higher biological functions [6]. *Ligilactobacillus salivarius* Li01 (CGMCC7045) has been shown to improve acute liver injury by inhibiting bacterial translocation, reshaping gut microbiota and increasing levels of proinflammatory cytokines [7, 8]. In our previous study, we demonstrated that intervention with Li01 is beneficial to the recovery of the gut microbiota and the intestinal barrier function in colitis mice [9]. These results indicate the potential anti-inflammatory effect of Li01, although its mechanism of action still remains unclear.

Food is not only the main source of energy for gut microbiota but also affects their composition and diversity. Diet therapy has recently become a popular method for treating chronic colitis [10]. Plant polyphenols, widely available in fruits and vegetables, usually contain unique anti-inflammatory properties and exhibit better effect than traditional carbohydrate prebiotics [11]. When lipophilic plant polyphenols are transported to the colon after oral administration, they can be fermented and utilized by the gut microbiota and are thus considered to be potential prebiotics [11]. The plant polyphenol resveratrol (RSV) is increasingly attracting attention as a promising prebiotic. Administration of RSV ameliorates inflammation by downregulating the relative levels of proinflammatory cytokines, enhancing the diversity of the microbiome and the abundance of *Lactobacillus* spp. and *Bifidobacterium* spp. in the gut microbiota of dextran sulfate sodium- (DSS-) induced colitis mice [12, 13]. In the gastrointestinal tract, RSV tends to be metabolized and form resveratrol-3-sulphate (RSV-sulfate), resveratrol-3-O-glucuronide (RSV-glucuronide), and dihydroresveratrol (DHR) [14–17]. Among these metabolites, DHR showed great bioactive effects for the treatment of diseases at a low dose [18]. Although many studies have investigated the treatment efficacy of either prebiotics or probiotics for colitis, plant polyphenols, as a potential prebiotic, can both promote the efficacy of probiotics and produce a significant therapeutic effect *via* synergistic interactions. The mechanism of action for this effect does, however, still need to be elucidated.

In the current study, we investigated the anti-inflammatory effects of RSV and Li01 combination in DSS-induced colitis mice. Intestinal barrier functions, immune factors, gut microbiota composition, and targeted and untargeted metabolomic profiles were determined after various interventions. The mechanism of enhanced anticolitis function was further explored by determining the interactions RSV and Li01, with a focus on RSV metabolism. In addition, the role of RSV and Li01 combination in interfering the aryl hydrocarbon receptor- (AHR-) and serotonin- (5-HT-) related pathways were also illustrated.

## 2. Results

### 2.1. Effects of RSV and Li01 Combination Treatment on Attenuating DDS-Induced Colitis in Mice

**Figure 1:**
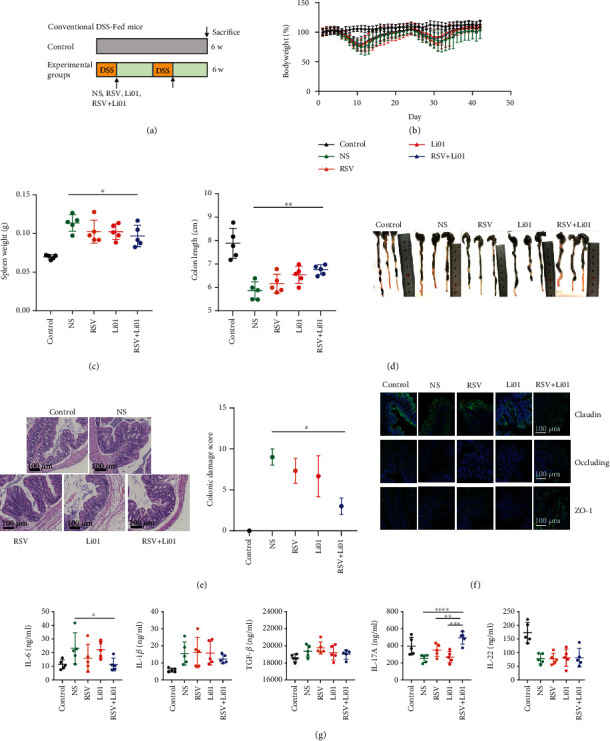
Therapeutic effect of RSV, Li01, and RSV+Li01 on DSS-induced colitis in mice. (a) Chart describing the experimental design in the DSS-induced colitis mouse model. DSS was dissolved in drinking water and fed on the 1^st^ and 4^th^ week. On the 2^nd^, 3^rd^, 5^th^, and 6^th^ week, mice in the experimental groups were orally administrated with PBS (NS), RSV, Li01, or RSV+Li01. (b–d) Body weight changes, spleen weight, and colon length were recorded and analyzed. (e) H&E staining and colonic damage scores for colon tissue of different groups. Scale bars: 100 *μ*m. (f) Expression of claudin, occluding, and ZO-1 in colon tissue evaluated by immunofluorescence. Scale bars, 100 *μ*m. (g) Concentration of proinflammatory and anti-inflammatory cytokines were analyzed in the serum. Data are presented as mean ± SEM, *n* = 5, ^∗^*P* < 0.05, ^∗∗^*P* < 0.01, ^∗∗∗^*P* < 0.001, and ^∗∗∗∗^*P* < 0.0001 for the comparison.

To examine the effects of RSV and Li01 on colonic inflammation, we administered DSS-fed male C57/BL6 mice with PBS, RSV, Li01, and RSV+Li01, separately (Figure 1(a)). The body weight of the mice was monitored daily. We found that mice fed with RSV+Li01 showed a slightly faster recovery compared to other groups (Figure 1(b)). Moreover, compared to the NS group, the colon was significantly longer, and the spleen weight was significantly decreased among mice fed with RSV+ Li01 (Figures 1(c) and 1(d)), indicating a better anti-inflammatory effect compared to other treatments. Histologic examination of colon sections (Figure 1(e)) revealed destruction of fewer crypt structures and less inflammatory cell infiltration, with a lower colonic damage score among mice in the RSV+ Li01 group compared to the NS group (*P* < 0.05), demonstrating that the combination of RSV and Li01 prevented inflammation from damaging the colonic epithelium. The barrier function was also evaluated through detection of tight junction-related proteins in the colonic mucosa. Immunofluorescence imaging (Figure 1(f)) showed that, compared to mice in the RSV and Li01 groups, mice in the RSV+Li01 group had an increased the expression of claudin, occluding, and ZO-1 proteins.

Fluctuations of the level of pro- and anti-inflammatory cytokines may be associated with the progression of colitis. We detected the serum level of proinflammatory cytokines, including interleukin IL-1*β*, IL-6, and transforming growth factor TGF-*β* as well as anti-inflammatory factors, including IL-17A and IL-22 (Figure 1(g)). Compared to mice in the control group, the concentration of IL-6 was elevated in all experimental groups except for the RSV+Li01 treatment. The levels of IL-1*β* and TGF-*β* were slightly decreased in the RSV+Li01 group compared to other colitis mice group but exhibited no statistical difference. Mice treated with RSV+ Li01 showed significantly increased concentration of IL-17A in the serum compared with other experimental groups (*P* < 0.0001), whereas for IL-22, mice in all experimental groups exhibited lower levels compared to the control group.

### 2.2. RSV and Li01 Combination Treatment Effect on Gut Microbiota Composition and Metabolism in DSS-Induced Colitis Mice

**Figure 2:**
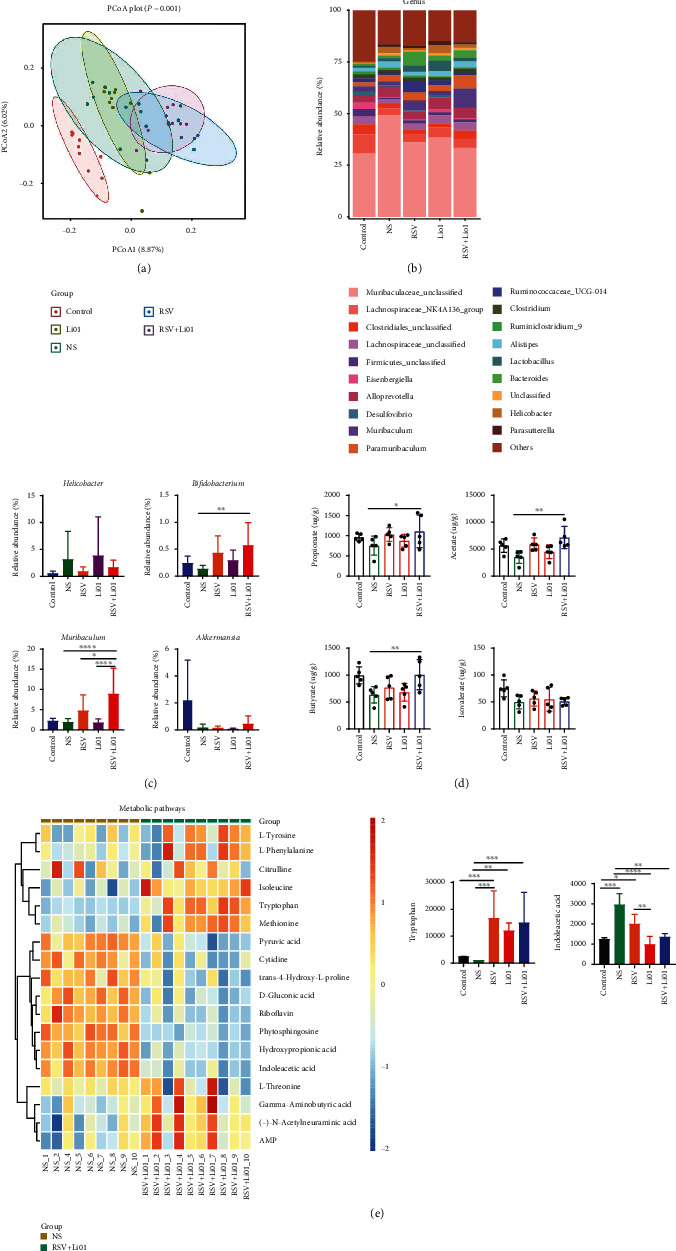
Alterations of gut microbiota and metabolic profiles in stool samples after different treatments in DSS-induced colitis mice. (a) *β*-Diversity of the gut microbiota by PCoA plots. (b, c) Relative abundance of microbial taxa at the genus level in each group. (d) Concentrations of SCFAs were measured (*n* = 5). (e) Analysis of various metabolites in metabolic pathway between NS and RSV+Li01 groups and the difference of tryptophan and indoleacetic acid concentration in all groups. Data are presented as mean ± SEM, ^∗^*P* < 0.05, ^∗∗^*P* < 0.01, ^∗∗∗^*P* < 0.001, and ^∗∗∗∗^*P* < 0.0001 for the comparison.

To explore the changes of the gut microbiota after intervention (PBS, RSV, Li01, or RSV+Li01), we used 16S rRNA gene sequencing to identify the composition of the gut microbiota in colonic content samples. Principal coordinates analysis showed that DSS-induced colitis mice in the RSV+Li01 group showed a significantly altered composition of their gut microbiota (*P* = 0.001, Figure 2(a)). The bacterial composition of mice in each group at the genus level was shown in Figures 2(b) and 2(c). The relative abundance of *Bifidobacterium* (*P* < 0.01), *Muribaculum* (*P* < 0.0001), and *Akkermansia* (*P* = 0.058) was significantly increased in mice fed with the RSV+Li01 combination compared to the NS group. The abundance of *Muribaculum* was also significantly higher in mice in the RSV+Li01 group compared to mice treated only with RSV or Li01. Compared to RSV and Li01 groups, the relative abundance of *Bifidobacterium* and *Akkermanisa* was slightly higher in RSV+Li01 group, with no significant difference. On the contrary, the abundance of *Helicobacter* tended to be lower in mice in the RSV+Li01 group compared to the NS group.

Since colitis is usually associated with decreased concentration of SCFAs in the colon, we measured the concentration of SCFAs in the feces samples by using gas chromatography-mass spectrometry (GC-MS). Compared to mice in the NS group, the levels of acetate, propionate, and butyrate were significantly increased in RSV+Li01 group, whereas for isovalerate, there was no statistically significant difference (Figure 2(d)).

The alteration of gut microbiota metabolism is a major cause of alleviating colitis; we then performed untargeted metabolomic analyses to identify the different fecal metabolite spectra between RSV+Li01 and NS groups (Figure S1a). As expected, the results showed a distinct difference between the metabolites of the two groups, of which most metabolites were annotated as lipids and lipid-like molecules as well as organic acids and derivatives. We showed all 56 metabolites of these two categories in which gamma-aminobutyric acid, L-threonine, methionine, L-phenylalanine, and endocannabinoids have been reported to exhibit anti-inflammatory effects. The relationship between the intestinal microbiota and metabolites was analyzed by using correlation heat map (Figure S1b). *Bifidobacterium* and *Muribaculum* were positively correlated with gamma-aminobutyric acid among mice in the RSV+Li01 group and negatively correlated with acylcarnitine among mice in NS group. To understand the functional characteristics of these differential metabolites, KEGG pathway enrichment analysis was conducted, and apparently, metabolic pathway was the dominant pathway enriched in the differential metabolite (Figure S1c). Among them, tryptophan observed to be elevated among mice in RSV+Li01 group compared to the NS group (Figure 2(e)). The levels of tryptophan among mice in the RSV, Li01, and RSV + Li01 groups were significantly higher than in control or NS groups. Additionally, indoleacetic acid, a metabolite of tryptophan, was observed to significantly increase among mice in the NS group.

### 2.3. Interactions between RSV and Li01

#### 2.3.1. Effect of RSV on the Growth of Li01

**Figure 3:**
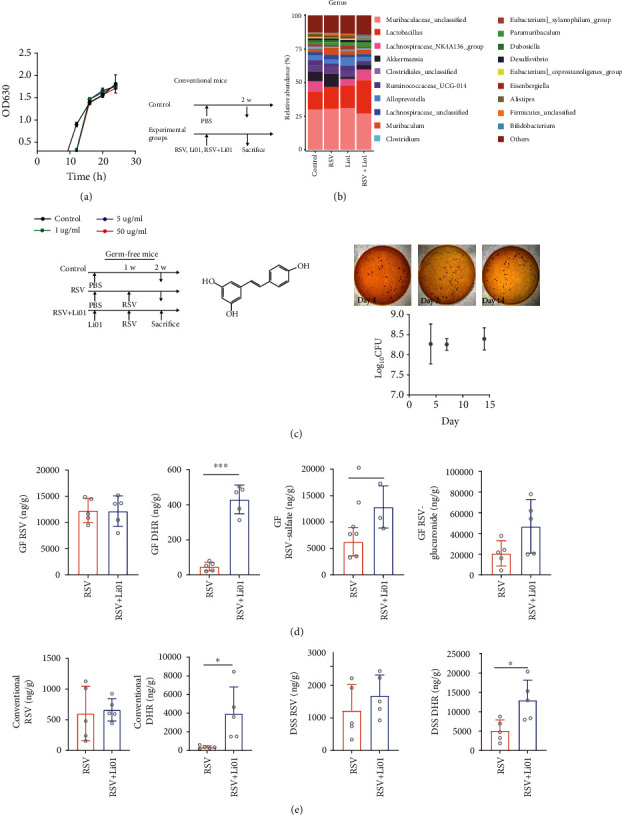
Interactions between RSV and Li01 through *in vivo* and *in vitro* studies. (a) Impact of RSV on the growth curve of Li01. (b) Experimental design for investigating the change in gut microbiota at the genus level of conventional mice subjected to different treatments, including RSV, Li01, and RSV+Li01. (c) Experimental design for studying the metabolism of RSV in GF mice before and after colonization with Li01. The Li01 colonies in feces collected on days 4, 7, and 14 in GF mice were shown. (d) Concentration of RSV and its metabolites were measured in feces of GF, conventional mice, and DSS-induced colitis mice (*n* = 5). Data are presented as mean ± SEM, ^∗^*P* < 0.05, ^∗∗^*P* < 0.01, ^∗∗∗^*P* < 0.001, and ^∗∗∗∗^*P* < 0.0001 for the comparison.

To better understand the mechanisms responsible for the synergistic effect of RSV and Li01 combination in DSS-induced colitis mice, *in vitro* and *in vivo* experiments were performed to clarify the RSV and Li01 interactions with each other. First, we determined the effect of RSV on the growth of Li01 *in vitro*. The effect of RSV on the growth of Li01 in culture medium was investigated. The results showed that the growth curves of Li01 did not change with the present of RSV in the culture media (Figure 3(a)).

Next, we evaluated how RSV interacts with Li01 *in vivo*. Conventional mice were fed with PBS, RSV, Li01, or RSV+Li01 by gavage daily for two weeks. Stool samples were collected and submitted for 16S rRNA gene sequencing. Compared to mice in the control and other experimental groups, the relative abundance of the genera *Lactobacillus* and *Bifidobacterium* in mice fed with RSV+Li01 was significantly elevated (Figure 3(b)). This result suggests that feeding with RSV promote the proliferation of *Lactobacillus* at the genus level, which may contribute to and facilitate the colonization of Li01.

We further investigated the effect of RSV on the growth of Li01 in a germ-free (GF) mice model. Mice were fed with Li01 and RSV according to the diagrammatic sketch in Figure 3(c). Feces were collected (on days 4, 7, and 14), and the bacterial colonies were detected. The results showed that Li01 was successfully colonized in the intestinal tract of mice, and ingestion of RSV did not significantly influence the concentration of Li01 in the intestinal tract (Figure 3(c)).

#### 2.3.2. Li01 Effect on the Metabolism of RSV

To determine the effect of colonized Li01 on the metabolism of RSV *in vivo*, RSV and its metabolites in fecal samples in the RSV and RSV+Li01 groups of GF mice were identified by liquid chromatography-tandem mass spectrometry (LC-MS/MS) analysis (Figure 3(d)). The metabolites were significantly elevated in the fecal samples after Li01 was colonized. Most RSV was metabolized into RSV-sulfate and RSV-glucuronide, with a small portion being metabolized to DHR. The concentration of DHR in the fecal samples of mice in the RSV+Li01 group was more than 120-fold higher compared to the RSV group. Interestingly, a similar trend for DHR could be observed among the conventional mice (Figure 3(e)) and DSS-induced colitis mice (Figure 3(f)). However, the amount of DHR was only a tenth of the concentration of RSV in the fecal samples in GF mice, whereas the ratio of DHR/RSV was increased to around 10 : 1 in fecal samples among both conventional and DSS-induced colitis mice, with the highest DHR/RSV ratio in colitis mice. In addition, RSV-glucuronide and RSV-sulfate in the fecal samples of conventional and DSS-induced colitis mouse models became undetectable, which could be influenced by the present of gut microbiota [18].

### 2.4. RSV and Li01 Combination Treatment Influencing the Environmental Sensor Mammalian Aryl Hydrocarbon Receptor (AHR)

**Figure 4:**
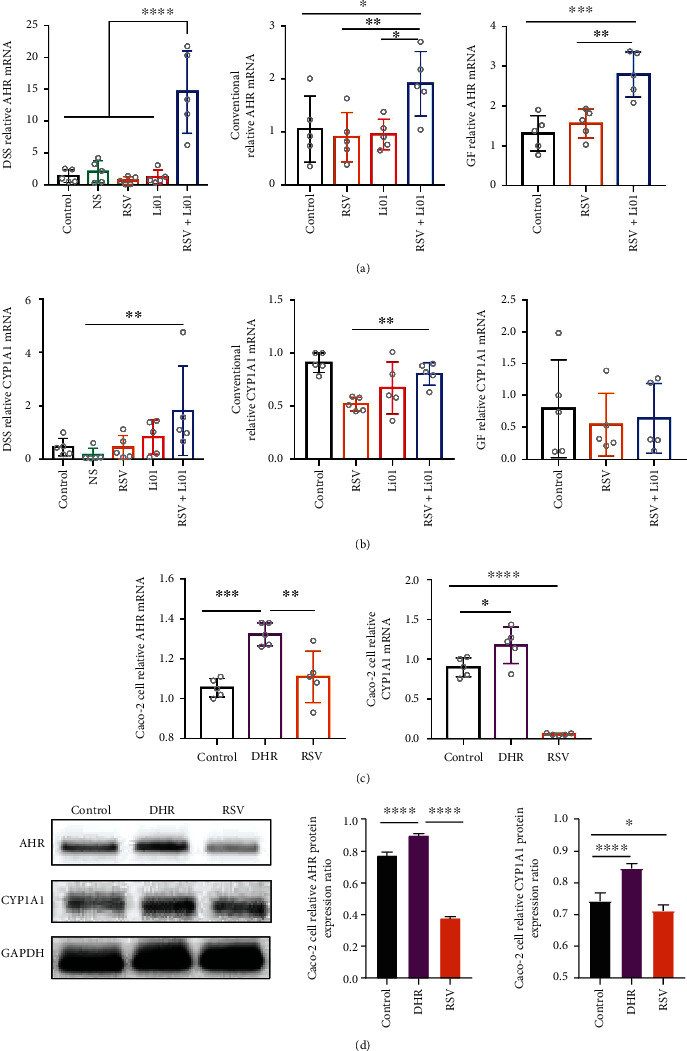
RSV and Li01 combination treatment improved intestinal barrier function by activating the expression of AHR. Relative mRNA levels of AHR (a) and CYP1A1 (b) in colon tissue of GF mice, conventional mice, and DSS-induced colitis mice were determined by RT-qPCR. Effect of RSV and DHR on the expression of AHR and CYP1A1 in Caco-2 monolayer cells by RT-qPCR analysis (c) and Western blot analysis (d). Data are presented as mean ± SEM, *n* = 5, ^∗^*P* < 0.05, ^∗∗^*P* < 0.01, ^∗∗∗^*P* < 0.001, and ^∗∗∗∗^*P* < 0.0001 for the comparison.

The environmental sensor mammalian aryl hydrocarbon receptor (AHR) is highly expressed in the gut, which can be activated by specific dietary components and mediates anti-inflammation function [19]. In this section, a significantly higher expression of AHR in the colonic tissue of DSS-induced colitis mice was found among mice in the RSV+Li01 group compared to any other groups (*P* < 0.0001, Figure 4(a)). Similar trend of AHR expression was shown in both GF mice and conventional mice (Figure 4(a)). Since the activation of AHR may induce the expression of the Ah-responsive gene CYP1A1 which is responsible for production of cytochrome P450 enzymes in the gut [20], thus, the level of CYP1A1 was also detected. The expression of CYP1A1 mRNA was found to be upregulated among mice in the RSV+Li01 group compared to other groups in three mouse models (Figure 4(b)). Since the introduction of Li01 in the gut microbiota significantly improved the level of the RSV metabolite DHR in all animal models, the direct effect of DHR on expression of AHR and CYPA1 was also determined by an *in vitro* study. As shown in Figure 4(c), DHR significantly increased the AHR mRNA in Caco-2 cell monolayers compared to the control (*P* < 0.001) and RSV groups (*P* < 0.01). Furthermore, DHR also upregulated the level of CYP1A1 expression compared to the control (*P* < 0.05) whereas RSV downregulated CYP1A1 mRNA level compared to the control group (*P* < 0.0001). Western blotting analysis also indicated higher protein levels of AHR and CYP1A1 in DHR group compared to control and RSV groups (Figure 4(d)).

### 2.5. Modulation of Serotonin Levels by RSV and Li01 Combination Treatment

**Figure 5:**
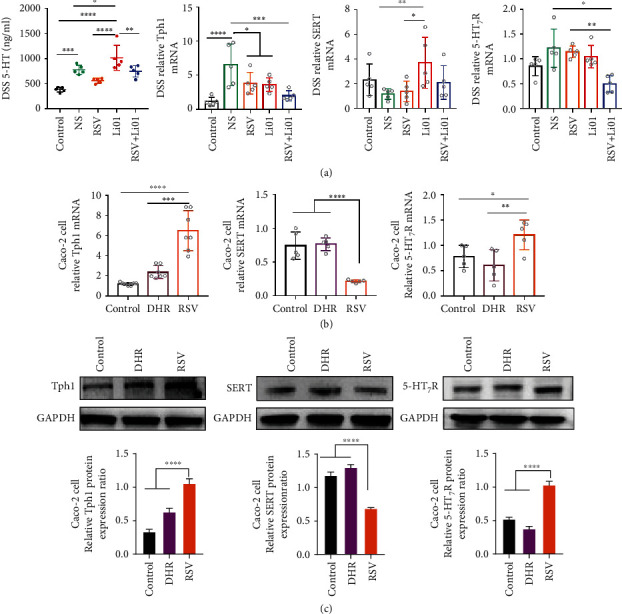
Impact of RSV and Li01 combination treatment on the serotonergic pathway in DDS-induced colitis mice. (a) 5-HT concentration in serum was quantified. The expression of Tph1, SERT, and 5-HT_7_R in the colon of DSS-induced colitis mice were analyzed by RT-qPCR. The expression of Tph1, SERT, and 5-HT_7_R in Caco-2 monolayer cells were determined by RT-qPCR analysis (b) and Western blot analysis (c). Data are presented as mean ± SEM, *n* = 5, ^∗^*P* < 0.05, ^∗∗^*P* < 0.01, ^∗∗∗^*P* < 0.001, and ^∗∗∗∗^*P* < 0.0001 for the comparison.

Serotonin (5-hydroxytryptamine, 5-HT) tends to increase in DSS-induced colitis mice, which can stimulate production of proinflammatory cytokines and accelerate the pathogenesis of colitis [21, 22]. Enterochromaffin cells are the main place for 5-HT production, which is mainly mediated by tryptophan hydroxylase 1 (Tph1) [23, 24]. Therefore, the serum level of 5-HT, expression of Tph1, serotonin reuptake transporter (SERT), and the 5-HT_7_ receptor (5-HT_7_R) in the colon were detected in this section (Figure 5(a)). The levels of 5-HT were significantly elevated among mice in the NS and Li01 groups compared to control group (*P* < 0.001). Compared to mice in the Li01 group, the levels of serum 5-HT were depressed among mice in the RSV and RSV+Li01 groups (*P* < 0.001). We found that the expression of Tph1 significantly upregulated among mice in the NS group compared to control group (*P* < 0.0001). Lower levels of Tph1 mRNA expression were observed among mice in the RSV, Li01, and RSV+Li01 groups compared to the NS group, with mice in the RSV+Li01 group showing the lowest levels (*P* < 0.001). SERT mRNA expression was elevated in the Li01 group compared to other groups. Regarding the expression of 5-HT_7_R, it was decreased in RSV+Li01 group, compared with NS, RSV, and Li01 group.

The effects of RSV and its metabolite DHR on the expression of Tph1, SERT, and 5-HT_7_R were evaluated in a Caco-2 cell monolayer model. The results in Figure 5(b) showed that RSV significantly upregulated the mRNA expression of Tph1 (*P* < 0.0001) and 5-HT_7_R (*P* < 0.05) whereas reduced the level of SERT (*P* < 0.0001) compared to control and DHR groups. DHR did not influence the expression of Tph1 or SERT, but slightly lowered the level of 5-HT_7_R expression compared to the control. The protein levels obtained by Western blot analysis were consistent with the results analyzed by RT-qPCR (Figure 5(c)).

In addition, the change of Tph1, SERT, and 5-HT_7_R after different treatments in conventional and GF mouse models were also evaluated. In conventional mice, the RSV and Li01 combination had the effect of increasing the serum level of 5-HT and the expression of Tph1, whereas the expression of 5-HT_7_R was decreased compared to all the other groups (Figure S2a). RSV+ Li01 also improved the level of Tph1 compared to other groups in GF mice (Figure S2b). Moreover, the concentration of 5-HT was significantly lowered when treatment with RSV alone compared to RSV + Li01 and control groups (*P* < 0.01).

## 3. Discussion

In this study, we established DDS-induced colitis mouse model by using C57/BL6 conventional mice. This model is widely used and simulates a system with many similarities with human IBD. Furthermore, the gut microbiota is essential for the development of robust colitis [25]. Previous research has shown that dietary RSV attenuated the inflammatory status and alleviated gut microbiota dysbiosis in colitis mouse models [13]. Based on the same model, oral administration of Li01 was found to facilitate intestinal barrier recovery and restoration of the gut microbiota [9]. The purpose of our study was to demonstrate the synergistic effect of coadministration of RSV and Li01 on regulating gut microbiota and alleviating colitis. The results shown in Figures 1(a)–1(f) indicated that colonic inflammation in mice treated with RSV+Li01 significantly attenuated compared to mice in the NS group in terms of lower spleen weight, longer colon, lower colonic damage score, and more favorable barrier function. However, the anti-inflammation effect was not obviously observable when RSV or Li01 were singly administrated. A similar combination treatment effect could be observed for serum inflammatory cytokines among mice in the RSV+Li01 group (Figure 1(g)); the levels of proinflammatory cytokines were decreased, whereas the levels of anti-inflammatory cytokines were elevated. Multiple studies have shown that IL-1 *β* and TGF-*β* are involved in the development of colonic inflammation and promote the expression of proinflammatory factor IL-6 [26, 27], whereas IL-17A and IL-22 are important regulators that influence inflammation and infection and regulate the composition of the microbiome [28].

Inflammation may cause impact on structure of the gut microbiota and the bacterial diversity [29]. We investigated the alteration in gut microbiota composition and metabolism after different interventions. The relative abundance of *Bifidobacterium*, *Akkermansia*, and *Muribaculum* were higher, whereas *Helicobacter* spp. were lower in mice fed with the RSV+Li01 combination compared to mice in the NS group (Figures 2(b) and 2(c)). *Helicobacter* spp. are positive to colonic inflammation [30]. However, *Akkermansia* and *Bifidobacterium* are promising probiotics for restoring the intestinal mucosal barrier and alleviating colitis [31, 32]. *Muribaculum* is a genus of the family *Muribaculaceae* which has been reported to be greatly decreased in mice with colitis progression [9]. These results indicated that combination treatment reversed the dysbiosis of gut microbiota caused by colitis. Compared to mice in the RSV and Li01 groups, combination treatment significantly elevated the abundance of *Muribaculum* (*P* < 0.05) and slightly increased the abundances of *Akkermansia* and *Bifidobacterium* although there was no statistical difference, which demonstrated combination treatment was better than RSV or Li01 treatment alone. Moreover, single RSV or Li01 has been demonstrated to alter the composition of the gut microbiota [9, 33]. *Muribaculum* and *Paramuribaculum* have been shown to be associated with SCFAs production, such as propionate and butyrate [34, 35]. In this regard, the increased abundance of *Muribaculum* was consistent with higher SCFA concentrations among mice in the combination group. Many studies have demonstrated that colonic inflammation is associated with decreased concentrations of SCFAs in the colon, resulting in decreased expression of intestinal tight junction proteins and higher expression of proinflammatory factors such as IL-1*β* and IL-6 [36–38]. Although previous experiments have indicated that both RSV and Li01 have anti-inflammatory effects on DSS-induced colitis mice [9, 13], our results showed that their function to stimulating the production of SCFAs was quite limited when they were used separately. Administration of the RSV and Li01 combination significantly improved the level of SCFAs, especially for propionate, acetate, and butyrate in the colon. Interestingly, both *Bifidobacterium* and *Muribaculum* were positively correlated with *γ*-aminobutyric acid among mice in RSV+Li01 group, whereas they were negatively correlated with acylcarnitine among mice in the NS group. *γ*-Aminobutyric acid, L-threonine, methionine, and L-phenylalanine have been reported to act as anti-inflammatory role in the development of inflammation by alleviating intestinal barrier damage, downregulating IL-1*β* and IL-6, or regulating immune function [39–42]. Acylcarnitine metabolites belonging to fatty acyls is usually correlated to activating the proinflammatory signaling pathway [43]. Therefore, our data demonstrated that the gut microbiota and associated gut metabolites may be one of the intermediates of synergistic effect which mediated combination treatment to improve colitis. Moreover, the level of tryptophan was relatively lower in mice with colitis, whereas RSV, Li01, and the RSV+Li01 combination treatments increased the concentration of tryptophan. For indoleacetic, it showed a reversed trend. These results indicated that the tryptophan metabolic pathway, which is thought to be closely related to disease status, may be altered [44]. Therefore, the regulatory effect of combination treatment on the tryptophan metabolic related 5-HT pathway was further determined in the following study.

In order to explore the mechanism of synergistic effect of RSV and Li01 combination in alleviating DSS-induced colitis in mice, the interactions between RSV and Li01 under different gut microbiota conditions were investigated. The addition of RSV did not affect the growth rate of Li01 compared to the control *in vitro*, while RSV and Li01 in combination significantly increased the abundance of *Lactobacillus* in conventional mice. Moreover, in the Li01 colonized GF mice, the introduction of RSV did not affect the concentration of cell count. A previous study has indicated that the regulatory effect of RSV on gut microbes is mainly mediated *via* its metabolites [45]. Thus, the probiotic effect of RSV on *Lactobacillus* spp. needs to be exerted through metabolism in the body with gut microbiota. More importantly, the synergistic effect may mainly lie not in the promotion of Li01 growth by RSV, but in the effect of Li01 on RSV metabolism *in vivo* after Li01 colonization. Due to the poor water solubility after oral administration, the majority of RSV will pass into the colon. The metabolism of RSV has been shown to be closely associated with the gut microbiota composition [46]. DHR is major microbiota-derived RSV metabolite [47]. The formed phase II metabolites are mainly glucuronide and sulfate conjugates, while RSV is metabolized primarily in the form of glucuronic acid conjugates [17]. Therefore, in the GF mouse model, RSV-glucuronide contributes to the highest portion in the fecal samples. The presence of Li01 accelerated the metabolism of RSV in the colon, as suggested by the dramatic increase in the concentration of DHR, RSV-sulfate, and RSV-glucuronide. Especially for DHR, its concentration in RSV+Li01-treated mice increased more than 120-fold compared with those in the RSV group. However, the portion of DHR was very low compared to RSV in the fecal samples. In the presence of gut microbiota, the levels of DHR were greatly elevated, with dramatically increased ratio of DHR/RSV in colitis mice, while RSV-sulfate and RSV-glucuronide were completely degraded. At the same time, these phase II metabolites will be further deconjugated in the gut, thus restoring RSV content [48]. In addition, the gut microbiota in DDS-induced colitis mice promoted much more RSV metabolized into DHR than that in conventional mouse. Since DHR dominated among mice in the colitis mouse model, we assumed it played a critical role in modulating the pathway related to colonic inflammation.

AHR, a nuclear receptor expressed on the intestinal surface, has been confirmed to function as a safeguarding barrier by influencing the expression of tight junctions [49]. AHR deficiency or inadequate AHR activation predisposes to induction of inflammatory disorders and inflammation-associated intestinal malignancy [50]. Activation of AHR induces expression of CYP1A1, a family of cytochrome P450 enzymes [19]. Figures 4(a) and 4(b) showed that in all three mouse models, the expression of both AHR were significantly increased in mice in the RSV+Li01 group compared to other groups, indicating AHR was simulated after combination treatment. The enhanced level of CYP1A1 also indicated the activation of AHR in DSS-induced colitis mouse model. According to the results from the *in vitro* cell study (Figures 4(b) and 4(c)), DHR was able to directly stimulate the expression of AHR and CYP1A1 while RSV severely inhibited the expression of CYPIA1. Therefore, it can be inferred that the activation of AHR was attributed to the increased ratio of DHR/RSV. In the DSS-induced colitis mouse model, the DHR/RSV ratio was significantly higher among mice treated with RSV+Li01 than mice in the RSV group, which may produce more DHR to activate AHR to suppress the inflammatory response and attenuate experimental colitis, while the ratio of DHR/RSV in GF mice was the lowest. Interestingly, there is absence of the AHR ligand in the gut of GF mice. Some *Lactobacillus* spp. are able to metabolize tryptophan to produce the ligand and stimulate AHR, which may explain the increased expression of AHR among mice in the RSV+Li01 group in GF mice [45]. Therefore, colonization of Li01 promoted production of DHR, an exogenous AHR ligand, which may be one of the mechanisms explaining the synergistic effect of RSV and Li01 in alleviating colitis. In addition, the significantly elevated level of IL-17A in Figure 1(g) can be also attributed to the activation of AHR [51]. IL-17A is a pluripotent and important cytokine in the development of colonic inflammation and hose defense [52].

According to the metabolomics data, microbiota tryptophan metabolism may be influenced after intervention with RSV, Li01, or Li01+RSV. The serotonin production pathway is the most important pathway of tryptophan metabolism, which is closely related to gut microbiota and intestinal inflammation [53]. Multiple studies have validated that 5-HT tends to be elevated in DSS-induced colitis mice, which may exacerbate the development of colitis [22, 54, 55]. The fluctuation of serum 5-HT was closely associated with the expression of Tph1 and SERT in the gut, since 5-HT is produced by Tph1 in enterochromaffin cells and transported to the blood vein by SERT [56]. Although the results shown in Figure 5(a) indicated that the serum levels of 5-HT were significantly higher in both mice in the NS and Li01 group compared to mice in the control group, they are based on different pathways. For NS group, the increased serum concentration of 5-HT was due to significantly increased expression of Thp1, while the level of SERT did not change, whereas the expression of SERT significantly elevated in Li01 group and the level Tph1 remained unchanged (Figure 5(a)). Interestingly, the level of 5-HT declined in the groups where RSV was introduced, including RSV and RSV+Li01 groups. Results from the *in vitro* experiments could possibly explain this; RSV could increase the expression of Tph1 while inhibiting the expression of SERT, leading to 5-HT being restricted in the intestinal lumen. In addition, although DHR did not appear to alter the levels Tph1 and SERT, it caused a reduction of the level of 5-HT_7_R. The 5-HT_7_R was also significantly reduced in colitis mouse model after treatment with the RSV and Li01 combination (*P* < 0.01). 5-HT usually works by activating specific 5-HT receptors family, among which the 5-HT_7_R has been confirmed to be significantly increased after induction of colitis in mice [57]. Therefore, reduced levels of 5-HT_7_R among mice in the RSV+Li01 group may predict ameliorated colitis. In conventional and GF mice models, we found that the RSV and Li01 combination had little effect on the serotonin pathway and might even slightly promote 5-HT synthesis. In a healthy state, the main function of 5-HT is to promote intestinal peristalsis and relieve constipation, which may explain the positive role of RSV and Li01 combination in conventional mice (Figure S2a) [58]. However, this requires further experimental verification. A lower ratio of DHR/RSV in conventional and GF mice may also limit the function in stimulating the serotonin pathway. In general, treatment with RSV or Li01 alone may also affect the serotonin pathway to contribute to the development of inflammation. However, in treating with RSV and Li01 combination, DHR will be a dominant metabolite which may also alter the expression of related molecules in the serotonin pathway to alleviate the colitis.

**Figure 6:**
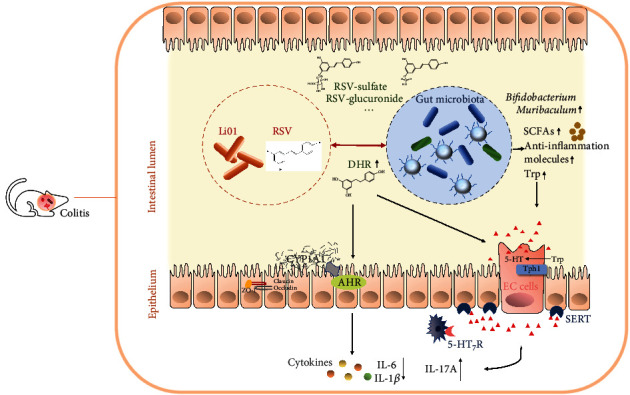
A schematic diagram illustrating the mechanism of synergistic effects of RSV and Li01 combination in treatment ameliorating DSS-induced colitis.

In summary, our results demonstrate that administering a combination of RSV and Li01 show promising therapeutic potential in treating colitis mice (Figure 6). Functioning as prebiotic and probiotic, RSV promoted the growth of Li01 which in turn affected the turn-over of RSV into more bioactive metabolites *in vivo*. Under the influence of Li01, DHR is a particularly dominant metabolite of RSV which can activate AHR and affect the serotonin pathway, facilitating the suppression of inflammation. These findings strengthen the case for using plant polyphenols in dietary interventions to enhance the function of probiotics in treating chronic diseases by facilitating biotransformation of beneficial metabolites from the gut microbiota. This study provides novel insights and suggests studies with a clinic focus are warranted to evaluate the potential intervention with plant polyphenol prebiotics in combination with probiotics to ameliorate IBD and as preventive strategies for other chronic inflammatory diseases. However, in this study, we did not confirm the enzymes which specifically transferred RSV into DHR. With regard to 5-HT pathway, we infer that RSV and Li01 combination may also associate with the gut-brain axis. Therefore, the relationships among DHR, gut-brain axis and colitis will be explored in further studies. In addition, a delivery system should be designed when this combination is applied in human studies.

## 4. Materials and Methods

### 4.1. Bacterial Propagation and Growth Conditions

Li01 was maintained in MRS broth (Oxoid, Basingstoke, Hampshire) enriched with 50% glycerol at -80°C. Li01 was anaerobically cultivated at 37°C in MRS broth for 24 h and maintained in the COY Vinyl anerobic chambers (Grass Lake, USA) with an airlock (95% N_2_ and 5% H_2_). Bacterial cells were collected after incubation for 24 h and diluted with saline buffer to obtain the concentration of about 10^9^ CFU/mL for further use.

### 4.2. Li01 Growth under RSV Exposure

Cis-RSV (Aladdin, Shanghai, China) was dissolved in the MRS broth to make different culture solution with concentration of 1 *μ*g/mL, 5 *μ*g/mL, and 50 *μ*g/mL, respectively. For control group, 100 *μ*L of Li01 stock solution was inoculated into 15 mL MRS broth and incubated in the anaerobic chamber under the temperature of 37°C for 24 h. Then, the bacterial solution was centrifuged, and the supernatant was discarded. The pellet was washed twice and resuspended to obtain constant volume with saline buffer. To investigate the influence of RSV on the growth of Li01, the bacterial concentration in the culture medium was measured at different time points (8 h, 12 h, 16 h, 20 h, and 24 h) by using the optical density (OD) value at 630 nm.

### 4.3. Experimental Design of *In Vivo* Mouse Models

#### 4.3.1. Set-Up of the DSS-Induced Colitis Mouse Model

Fifty specific pathogen-free (SPF) C57/BL6 mice (5-week-old, male) were purchased from Zhejiang Laboratory Animal Center (Hangzhou, China). They were fed with AIN93G diet (70 g of soybean oil/kg and 200 g of casein diet, Changzhou SYSE Bio-tec Co., Ltd., China). After acclimation for 3 days, mice were randomly divided into five groups (*n* = 10) designated as the control group, the negative (NS) group, the RSV group, the Li01 group, and the RSV+Li01 group. All mice were induced with chronic colonic inflammation by feeding them 3% DSS (MP Biomedicals, Shanghai, China) at weeks 1 and 4, except mice in the control group. Mice in experimental treatment groups were given with 200 *μ*L saline buffer containing 30 mg RSV or Li01 everyday on weeks 2, 3, 5, and 6 while mice in the control and NS groups were intragastrically administered with 200 *μ*L PBS. Weight was recorded daily during feeding. All mice were sacrificed on the 42^th^ day. Feces and serum samples were collected. The mice were dissected, the colon length was measured, and the distal part of colon was collected for immunofluorescence staining and histological assessment. Spleen tissues were weighed and colon contents were saved for 16S rRNA and metabolomic analysis. All the samples were stored at -80°C.

#### 4.3.2. Set-Up of the Conventional Mouse Model

Forty SPF C57/BL6 mice (5-week-old, male) were acclimated for 3 days according to the conditions described above. The mice were randomly divided into four groups (*n* = 10), designated as the control, RSV, Li01, and RSV+Li01 groups. Except the control group, of which mice were fed with 200 *μ*L saline buffer, the mice in other groups were fed intragastrically with 200 *μ*L saline buffer containing 30 mg RSV or Li01 for 14 days and sacrificed. Serum, feces, spleen, colon, and colonic content were collected and stored at -80°C freezer for further analysis as described above.

#### 4.3.3. Set-Up of the Germ-Free Mouse Model

Eighteen GF C57/BL6 mice (6-week-old, male) were bred and acclimated in a sterile isolator according to a previous method [59]. The mice were randomly divided into three groups (*n* = 6), including the control group, the RSV group, and the RSV+Li01 group. For control group, mice were intragastrically administered with 200 *μ*L saline buffer on the 1^st^ day and then fed with AIN93G diet for continuous 14 days and sacrificed on the last day. For RSV group, on the first day, mice were provided with 200 *μ*L saline buffer and fed with the AIN93G diet for 7 days. From the 8^th^ day, mice were fed with the AIN93G diet containing 500 ppm RSV (Changzhou SYSE Bio-tec Co., Ltd., China, RSV diet) for another 7 days and subsequently sacrificed on the 14^th^ day. Mice in the RSV+Li01 group were fed with 200 *μ*L Li01 solution (~10^9^ CFU/mL) by gavage on the first day and fed with the AIN93G diet for 7 days. From the 8^th^ day, the diet was changed into RSV diet before being sacrificed on the 14^th^ day. Feces were collected at 4, 7, and 14 days from mice in the RSV+Li01 group, to study the colonization of Li01. All mice were weighed before sacrifice, after which feces, serum, and colon samples were collected as described above.

### 4.4. Exposure of Caco-2 Cell Monolayers to RSV and DHR

Human intestinal Caco-2 cells were cultured in Dulbecco's modified Eagle's medium (DMEM, Gibco, Thermo Fisher Scientific, Waltham, USA) supplemented with 4.5 g/L glucose, 15% fetal bovine serum, 1% amino acids, and 1% penicillin-streptomycin in culture dishes. Cells were harvested when they grew to 90% confluence. Then, they were seeded at 5.5 × 10^5^ cells/mL on the 6-well polyester Transwell plates (Corning Inc., New York, USA). Before experimental treatment, the cells were cultured for 20-23 days to form monolayers. The passage number of cells used for experiments was between 20 and 40. Caco-2 monolayers were treated with or without DHR (150 *μ*M, Macklin Biochemical, Shanghai, China) and RSV (150 *μ*M) in DMEM medium for 24 h. After treatment, the transepithelial electrical resistance (TEER) of cell monolayers were measured. Then, the cell extracts were harvested for subsequent Western blot and RT-qPCR analysis.

### 4.5. Histological Assessment of Mouse Colon Tissues

The distal colon tissue was fixed with formalin overnight and then was embedded with paraffin. The biopsy has a section thickness of 5 *μ*m and was stained with haematoxylin and eosin (H&E). Colonic injury score was calculated according to the protocol described [60].

### 4.6. Immunofluorescence Imaging of Mouse Colon Tissue

The paraffin-embedded colon sections were deparaffinized and soaked in 0.01 M Tris/EDTA (Sigma-Aldrich, Saint Louis, USA) for 10 min. Then, the tissues were permeabilized by using 0.1% Triton X-100 (Sigma-Aldrich, Saint Louis, USA) and blocked by using PBS containing 1% BSA and 22.52 mg/mL of glycine. The samples were incubated overnight with the primary antibodies ZO-1 (1 : 500), occludin (1 : 500), and claudin (1 : 1000) at 4°C purchased from Abcam (Cambridge, UK). Then, the Cy3-conjugated goat anti-rabbit secondary antibody (Abcam) and DAPI (Life Technologies, Carlsbad, USA) were stained before they were visualized by a laser scanning confocal microscopy (Leica, Wetzlar, Germany).

### 4.7. Quantification of Serum Cytokines

Serum cytokines (IL-1*β*, IL-6, TNF-*β*, and IL-17A) in mice were detected by using a Bio-Plex Pro Mouse Cytokine 6-Plex Panel (Bio-Rad, Hercules, USA) on the MAGPIX system (Luminex, Austin, USA). The concentration of serum IL-22 and 5-HT was quantified by the Quantikine ELISA Kit (R&D Systems, Minneapolis, USA) and ELISA kit (Elabscience Biotechnology Co. Ltd., Houston, USA), respectively, according to the instructions.

### 4.8. DNA Extraction and 16S rRNA Gene Sequencing

DNA from bacteria of colon contents was extracted with the E.Z.N.A. ®Stool DNA Kit (OMEGA Bio-Tek Inc., Norcross, USA) as the manufacturer's protocols. The total DNA was stored at -80°C for subsequent analyses. The V3-V4 region of the 16S rRNA gene was amplified and purified using the same method as previously described [61]. Purified amplicons were further used to construct sequencing libraries, followed by sequencing on the Illumina NovaSeq platform (Illumina, San Diego, USA). Then, paired-end reads were generated through Trimmomatic software. Operational taxonomic units were generated by merging, denoising, and clustering qualified reads using QIIME software and Vsearch software. PCoA analysis was displayed using R software.

### 4.9. RT-qPCR

RNA from colonic tissues and cell extracts were extracted with the TRIzol reagent (Invitrogen, Carlsbad, USA). The PrimeScript RT Master MiX (TaKaRa, Kusatsu, Shiga, Japan) was used to transcribe RNA into cDNA. RT-qPCR was performed with the synthesized cDNA using a TB Green Premix Ex Taq II kit (TaKaRa, Kusatsu, Shiga, Japan). Sequences of the gene-specific primers of Tph1, AHR, CYP1A1, SERT, 5-HT_7_R, and GAPDH genes were displayed in Table S1. The relative mRNA levels were analyzed by method 2^−ΔΔCt^.

### 4.10. Western Blot Analysis

Proteins were extracted from Caco-2 cell monolayers by using a freshly prepared cell lysis buffer supplemented with 1 mM phenylmethylsulfonyl (PMSF) (Beyotime, Shanghai, China). The concentration of proteins was quantified using a BCA protein assay kit (Beyotime, Shanghai, China). The sample supernatants were diluted to a proper concentration and mixed with sodium dodecylsulfate polyacrylamide gel electrophoresis (SDS-PAGE) sample loading buffer (Beyotime, Shanghai, China) before being denatured (boiling for 5 min). Then, the samples were fractionated with SDS-PAGE and electrophoretically blotted onto polyvinylidene fluoride membranes (Beyotime, Shanghai, China). The membranes were immersed in Quickblock blocking buffer (Beyotime, Shanghai, China) for 1 h and then incubated with primary antibodies of 5HT_7_R (1 : 1000), AHR (1 : 1000), Tph1 (1 : 500), SERT (1 : 1000), CYP1A1(1 : 1000), or GAPDH (1 : 2500) at 4°C overnight. These antibodies were purchased from Abcam. Membranes were washed before being incubated with the secondary antibody (Genscript, Piscataway, USA) for 1 h at room temperature. After 3 × 10 min washing in TBS-Tween-20, the bands were detected by using an ECL detection kit (Beyotime, Shanghai, China).

### 4.11. Extraction of Fecal Samples and LC-MS/MS Analysis

20 *μ*L of fecal samples were mixed with 50 *μ*L of tolbutamide as internal standard and then rotated at 3,900× g for 10 min. Subsequently, 200 *μ*L supernatant was diluted with 200 *μ*L solution (acetonitrile : water = 1 : 1, *v*/*v*) before analyzed by LC-MS/MS. The recovery rate of RSV and metabolites extraction was above 90% for fecal samples. The MS analysis was performed on the LTQ-Orbitrap mass spectrometer (Thermo Fisher Scientific, Bremen, Germany) with a negative ionization mode of electrospray ionization. Venvsil MP C18 column (2.1 mm × 100 mm, 3.5 *μ*m) was used for separation. Solvent A and solvent B were MNH_4_OAC/FA (100/5, *v*/*v*) and acetonitrile separately, with a flow rate set to 0.6 mL/min. The gradient was as follows: 45% B liner (0-0.4 min), 85% B liner (1.0 min), 85% B liner (1.0-3.0 min), and 45% B liner (3.2 min). Other conditions were as follows: injection volume = 15 *μ*L, column temperature = 40°C. The MS detector was set to the multiple reaction monitoring mode by using the quantification ions *m*/*z* 227 ([M-H]-) → 185 ([M-H]-); 307 ([M-H]-) → 185 ([M-H]-); 403 ([M-H]-) → 227 ([M-H]-); and 229 ([M-H]-) →123 ([M- H]-) for RSV, RSV- sulfate, RSV-glucuronide, and DHR, respectively [62]. The linearity was in the range of 50-50000 ng/g, and the lowest limit of qualification was 50 ng/g in feces samples. RSV and its metabolites were quantified according to a previous method [62].

### 4.12. Quantification of SCFAs

Fecal samples (50 mg) were extracted with 500 *μ*L methanol-water solution which contained 0.1% HCl and 20% H_2_O. Samples were added acetic acid-D4, crushed by using a freezing crusher and ice bath ultrasound for 10 min before centrifugation. 200 *μ*L upper liquid in the sample bottle was used for GC-MS analysis according to a previous reported method [63]. An Agilent HP-Innowax GC column (length: 30 m, ID: 0.25 mm, film thickness: 0.25 *μ*m) was employed with 1 *μ*L injection volume without split columns. Helium was used as the carrier gas. The injection port temperature is set at 240°C. The column temperature was set to 50°C for 1 min and increased to 180°C with a rate of 10°C/min and then increased to 240°C at a rate of 40°C/min and held for 3 min. Mass spectrometry was performed at an EI voltage of 70 eV. The retention time and SIM fragment ions of SCFAs were qualitatively compared with internal standards. The MS spectra data collection and quantitative analysis of target compounds were completed by Xcalibur (Thermo Fisher Scientific, Waltham, USA).

### 4.13. Metabolomics Profiling of Fecal Samples

#### 4.13.1. Metabolite Extraction and Metabolomics Analysis by LC-MS/MS

The fecal samples were thawed on ice, and the 20 *μ*L samples were extracted with 120 *μ*L precooled 50% methanol buffer and then stored at -20°C. The supernatant was collected for further LC-MS analysis. In addition, 10 *μ*L extract was mixed for preparing QC samples. A Triple TOF 5600 Plus high-resolution tandem mass spectrometer (SCIEX, Warrington, UK) was used for the analysis of all the extracted samples. Chromatographic separation and reversed-phase separation were performed according to a previous described method [64]. The mobile phase consisted of solvent A (water, 0.1% formic acid) and solvent B (acetonitrile, 0.1% formic acid) and delivered at a flow rate of 0.4 mL/min. We used the same gradient elution conditions as described previously [65]. The metabolites eluted from the column were detected by using the Triple TOF 5600 Plus system. Each sample was collected in positive ion mode and negative ion mode. We monitored the 40 GHz detector with four-anode/channel and summed the four-time bins for each scan with a frequency of 11 kHz and converted them into data. During the acquisition process, mass accuracy was corrected at intervals of 20 samples, and the QC product was analyzed every 10 samples to evaluate the stability of the LC-MS.

#### 4.13.2. Metabolomics Data Processing and Analysis

Metabolites were annotated by matching the exact molecular mass data (*m*/*z*) of samples with those from database of online KEGG and HMDB. The relative standard deviations of metabolic features were calculated, and samples with a standard deviation less than 30% were reserved for further analysis of data. Before analysis, the group datasets were normalized. Analysis was performed by using the metaboanalyst.ca online tool [66].

### 4.14. Statistical Analysis

The results were presented as means ± SEM. One-way ANOVA followed by Fisher's LSD post hoc test (more than two groups) or Student's *t*-test (two groups) was used for statistically significant (^∗^*P* < 0.05, ^∗∗^*P* < 0.01, ^∗∗∗^*P* < 0.001, and ^∗∗∗∗^*P* < 0.0001). All statistical analyses were performed using GraphPad Software (version 7.0) and R software (version 4.1.2).

## Data Availability

All the data in this study are presented in the paper or the Supplementary Materials. Additional data relevant to the article are available from the corresponding author upon request.

## Supplementary Materials

Supplementary Materials Figure S1: altered fecal metabolomics and gut microbiota in RSV+Li01 group compared with NS group in DSS-induced colitis mice. (a) Heat map of hierarchical cluster analysis showing the 56 differential metabolites between RSV+Li01 group and NS group. The blue boxes indicate a lower-than-average expression ratio, and the red boxes indicate a higher-than-average expression ratio. A shorter Euclidean distance of the tree clusters implies higher similarity between two substances. (b) Pearson correlation analysis of the association of the gut microbes and top 20 differential metabolites in RSV+Li01 group and NS group. (c) Pathway enrichment analysis of differential metabolites according to the KEGG pathway between RSV+Li01 group and NS group. ^∗^*P* < 0.05, ^∗∗^*P* < 0.01. Figure S2: impact of RSV and Li01 combination treatment on the serotonergic pathway in conventional (a) and GF (b) mice. 5-HT concentration in serum was quantified. The expression of Tph1, SERT, and 5-HT7R were analyzed by RT-qPCR in the colon of conventional and GF mice. Data are presented as mean ± SEM, *n* = 5, ^∗^*P* < 0.05, ^∗∗^*P* < 0.01, ^∗∗∗^*P* < 0.001, and ^∗∗∗∗^*P* < 0.0001 for the comparison. Table S1: sequences of the gene-specific primers.
