# Supplementary material for: The Role of Dihydroresveratrol in Enhancing the Synergistic Effect of Ligilactobacillus salivarius Li01 and Resveratrol in Ameliorating Colitis in Mice
Source: Research (Wash D C). 2022 Jun 14;2022:9863845. doi: 10.34133/2022/9863845 (PMC9275091; doi:10.34133/2022/9863845)
Supplement: Supplementary Materials — Figure S1: altered fecal metabolomics and gut microbiota in RSV+Li01 group compared with NS group in DSS-induced colitis mice. (a) Heat map of hierarchical cluster analysis showing the 56 differential metabolites between RSV+Li01 group and NS group. The blue boxes indicate a lower-than-average expression ratio, and the red boxes indicate a higher-than-average expression ratio. A shorter Euclidean distance of the tree clusters implies higher similarity between two substances. (b) Pearson correlation analysis of the association of the gut microbes and top 20 differential metabolites in RSV+Li01 group and NS group. (c) Pathway enrichment analysis of differential metabolites according to the KEGG pathway between RSV+Li01 group and NS group. ∗P < 0.05, ∗∗P < 0.01. Figure S2: impact of RSV and Li01 combination treatment on the serotonergic pathway in conventional (a) and GF (b) mice. 5-HT concentration in serum was quantified. The expression of Tph1, SERT, and 5-HT7R were analyzed by RT-qPCR in the colon of conventional and GF mice. Data are presented as mean ± SEM, n = 5, ∗P < 0.05, ∗∗P < 0.01, ∗∗∗P < 0.001, and ∗∗∗∗P < 0.0001 for the comparison. Table S1: sequences of the gene-specific primers. [file 9863845.f1.docx]

**Supplementary Materials**

**Figure S1**


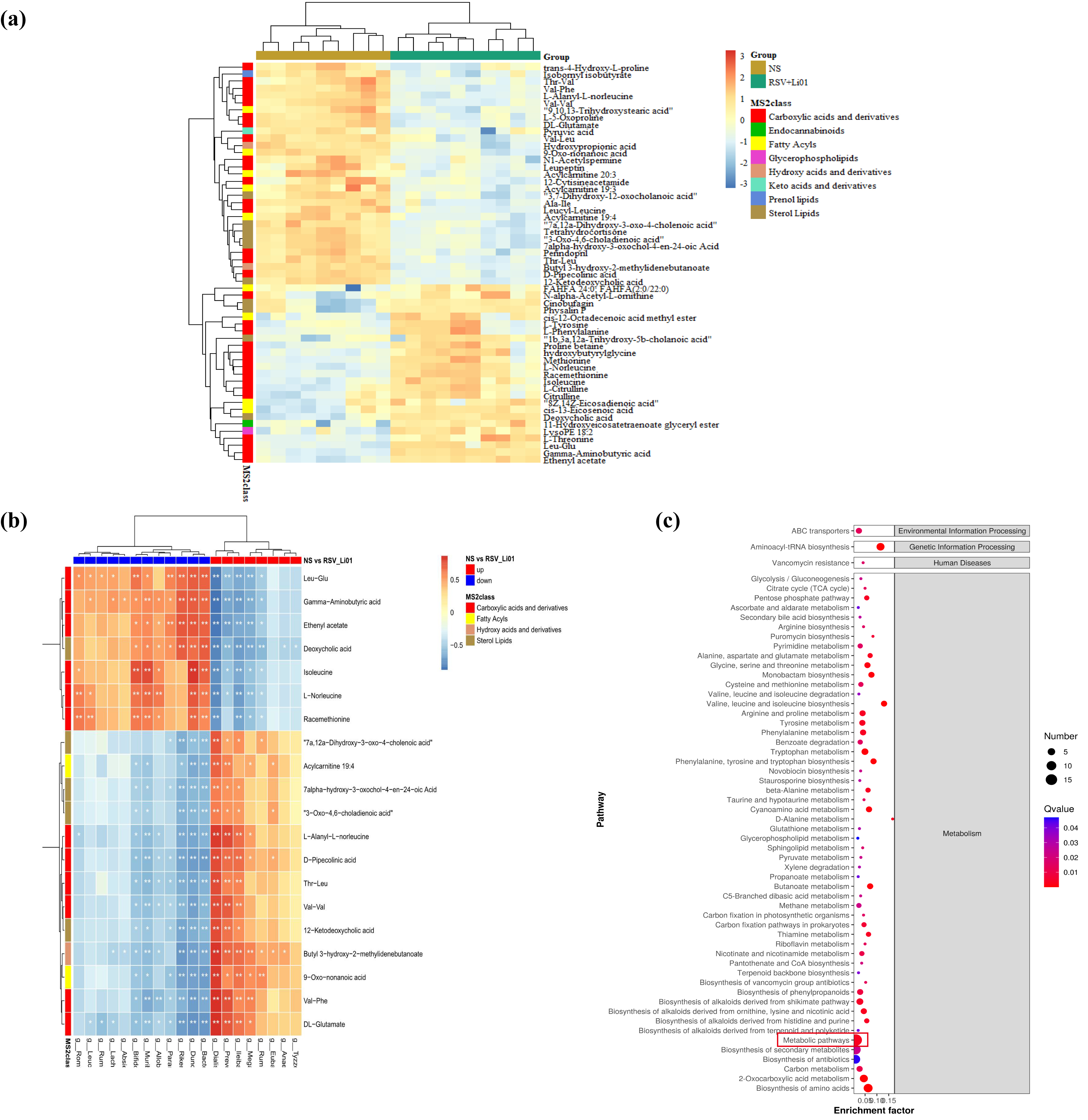


**Figure S2**


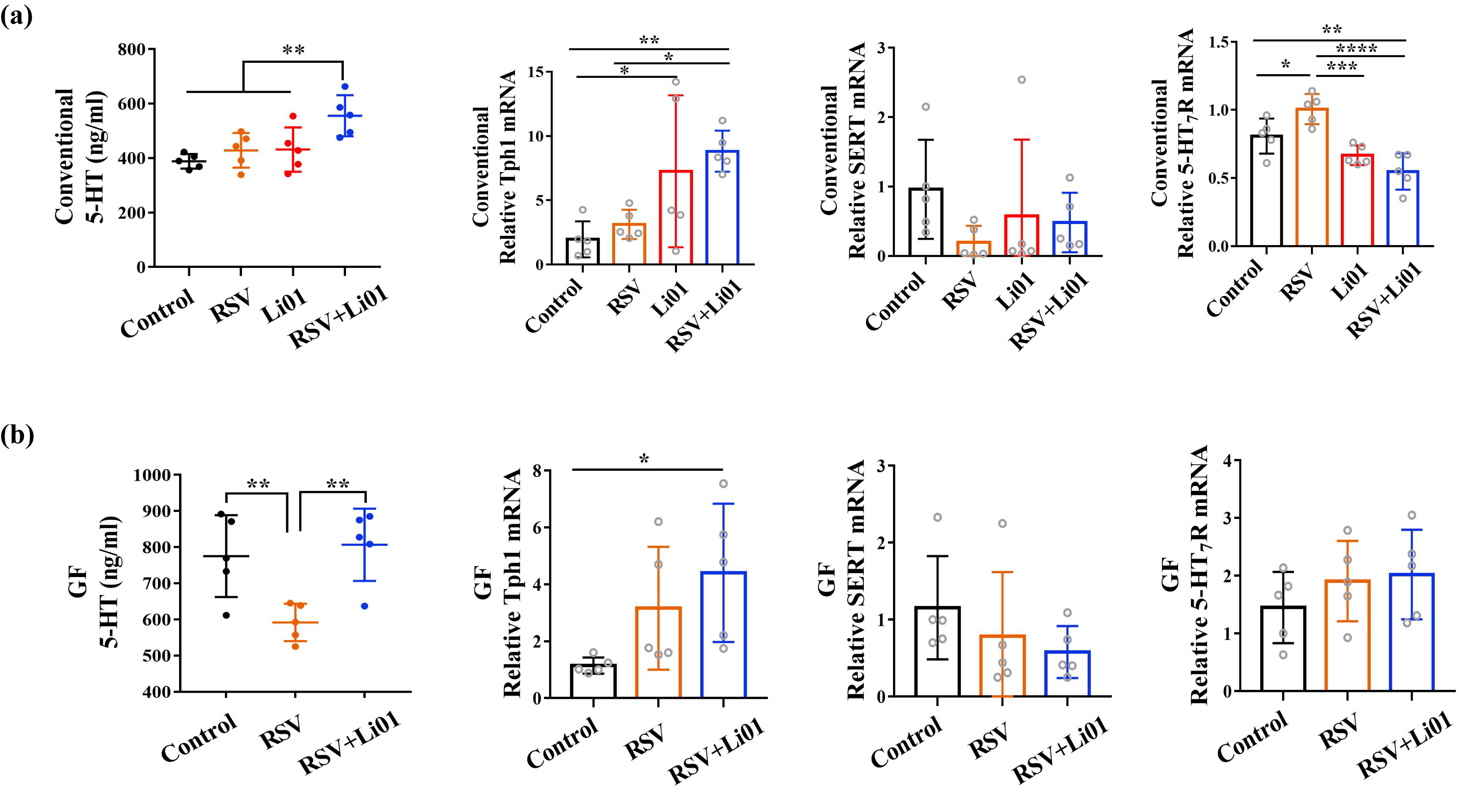


**Table S1. Sequences of the gene-specific primers**

| Gene | Species | Forward primer (5′→3′) | Reverse primer (5′→3′) |
| --- | --- | --- | --- |
| GAPDH | Mouse | CAGTGGCAAAGTGGAGATTGTTG | TCGCTCCTGGAAGATGGTGAT |
| Tph1 | Mouse | AAGAAATTGGCCTGGCTTC | GTTTGCACAGCCCAAAACTC |
| AHR | Mouse | GAGGTGGGTCCAGTCCAATG | GCCTCTCCGGTAGCAAACAT |
| 5-HT_7_R | Mouse | GTCATCTCCCTGAATGGCGT | GTGAAGGCTCCCACGATGAT |
| SERT | Mouse | GCGACGTGAAGGAAATGCTG | GGAGTTGGGGTGGACTCATC |
| CYP1A1 | Mouse | TTCTCCATAGCCTCGGACCC | ACATTGGCTACTGACACGACC |
| GAPDH | Human | GGAGTCCACTGGCGTCTTCA | GTCATGAGTCCTTCCACGATACC |
| Tph1 | Human | CGTCCTGTGGCTGGTTACTT | CCTCTGAAGCGCCAAGAGAA |
| AHR | Human | ACAACCGATGGACTTGGGTC | TGGCAGGAAAGGGTTGGTT |
| 5-HT_7_R | Human | CTCCGCCTCCATCACCTTAC | GTGTTTGGCAGCACTCTTCC |
| SERT | Human | GACCTTGCTTGCCCTCTCTT | TTTGTGGATCACCTCCGAGC |
| CYP1A1 | Human | TAATTTCGGGGAGGTGGTTGG | TGTCTGTGATGTCCCGGATG |
